# Association between clinical parameters and size of three-dimensionally reconstructed anatomical abnormalities in patients with lateral epicondylitis: a cross-sectional study

**DOI:** 10.1186/s13018-021-02406-5

**Published:** 2021-04-26

**Authors:** Seok Woo Hong, Jeong-Hyun Kang, Jin Hun Park, Ji Na Kim, Hee Jin Park, Eugene Kim

**Affiliations:** 1 Department of Orthopedic Surgery, Kangbuk Samsung Hospital, Sungkyunkwan University School of Medicine, 29, Saemunan-ro, Jongno-gu, Seoul, 03181 Republic of Korea; 2 Clinic of Oral Medicine and Orofacial Pain, Institute of Oral Health Science, Ajou University School of Medicine, 164, Worldcup-ro, Yeongtong-gu, Suwon, Gyeonggi-do 16499 Republic of Korea; 3 Department of Radiology, Kangbuk Samsung Hospital, Sungkyunkwan University School of Medicine, 29, Saemunan-ro, Jongno-gu, Seoul, 03181 Republic of Korea

**Keywords:** Lateral epicondylitis, Common extensor tendon, Three-dimensional reconstruction, Thresholding technique, Magnetic resonance imaging

## Abstract

**Background:**

The association of the severity of clinical symptoms and level of functional performance with the degree of magnetic resonance imaging abnormalities in patients with lateral epicondylitis has not been fully elucidated. This study aimed to investigate the association between the degree of anatomical abnormalities by evaluating three-dimensional magnetic resonance imaging models of the common extensor tendon and clinical parameters in patients with lateral epicondylitis.

**Materials and methods:**

A total of 61 patients (24 men and 37 women) with lateral epicondylitis were included in this study. 3-Tesla magnetic resonance imaging was performed for all patients, and clinical parameters, including pain visual analog scale score, Quick Disabilities of Arm, Shoulder and Hand questionnaire score, elbow range of motion, and demographic factors, were evaluated. The proportion of lesion volume of common extensor tendon was adopted for three-dimensional model analysis. To determine the factors associated with clinical parameters, univariate, and multivariate linear regression analyses were performed.

**Results:**

The proportion of lesion volume of common extensor tendon was not associated with clinical parameters. Gender and muscle edema were independently associated with pain visual analog scale scores. However, demographic factors and magnetic resonance imaging abnormalities were not associated with the Quick Disabilities of Arm, Shoulder, and Hand questionnaire score or elbow range of motion.

**Conclusions:**

The three-dimensional volumetric lesion size of common extensor tendon was not associated with clinical symptoms and functional performance in patients with lateral epicondylitis. The clinical parameters of lateral epicondylitis may be influenced by several factors.

## Introduction

Lateral epicondylitis, commonly referred to as tennis elbow, is one of the most common disease that causes pain in the lateral side of the elbow and upper extremity functional limitation [1]. The incidence rate of lateral epicondylitis was 3.4 per 1000 person-years, and women had slightly higher incidence rate than men [2, 3]. It is known to be caused by degeneration of the common extensor tendon originating from the lateral epicondyle of the humerus [4, 5] and can usually be diagnosed on the basis of clinical symptoms and physical examination results [6]. In addition, magnetic resonance (MR) imaging could be taken into consideration, particularly in patients who need surgical treatment, to confirm the presence of lesions in the common extensor tendon and its associated pathologies [7].

In the early stage of lateral epicondylitis, conservative treatments such as stretching exercise and counterforce brace application can be performed. If clinical symptoms do not improve or even worsen after sufficient conservative treatment, surgery can be considered. Generally, compared with the patients who receive only conservative treatment, those who undergo surgical treatment might suffer from a greater degree of pain, a lesser degree of elbow range of motion (ROM), and more severe common extensor tendon degeneration [8]. Previous studies already showed the relationships between the sizes of common extensor tendon abnormalities measured using two-dimensional (2D) imaging modalities and clinical symptoms [9, 10]. Owing to imaging distortion and superimposition of surrounding structures, accurate evaluation of the condition of common extensor tendon abnormalities using 2D analyses is challenging.

Recently, with the development of three-dimensional (3D) graphic processing technology, programs to reconstruct 2D image data into 3D models have been widely used in routine clinical practice [11]. Not only computed tomography (CT) images, but also MR images can be reconstructed in three dimensions. Particularly, the soft tissues, primarily evaluated in MR imaging, can be reconstructed and analyzed using 3D models. However, to the best of our knowledge, there were no studies in the literature which have investigated the relationship between the size of common extensor tendon abnormalities measured using reconstructed 3D models and clinical parameters.

Therefore, this study aimed to investigate the relationships between the degree of anatomical abnormalities of common extensor tendon using 3D reconstructed MR images, and severity of clinical symptoms and level of functional performances in patients with lateral epicondylitis.

## Materials and methods

### Participants

A total of 61 patients (24 men and 37 women) with lateral epicondylitis from April 2014 to March 2020 were included in this retrospective study. MR imaging data, plain radiographic series, and clinical records of the patients were assessed. The inclusion criteria were as follows: (1) patients who were clinically diagnosed with unilateral lateral epicondylitis with contralateral normal elbow joint, (2) patients who underwent 3-Tesla MR imaging, and (3) patients who suffered from related clinical symptoms more than 3 months before undergoing MR imaging. The exclusion criteria were as follows: (1) patients with a history of corticosteroid injection within 3 months of undergoing MR imaging, (2) patients with a history of trauma to the elbow joint, (3) patients who were diagnosed with degenerative arthritis or inflammatory arthritis in the affected side of the elbow, and (4) patients associated with worker’s compensation such as private or public insurance. The following demographic and clinical data were compiled using an electronic medical record system: age at the time of undergoing MR imaging, affected side, gender, body mass index (BMI), self-assessment pain visual analog scale (VAS) score, Quick Disabilities of Arm, Shoulder, and Hand (QuickDASH) questionnaire score, and elbow ROM.

### MR imaging examinations of 2D image data

A 3-Tesla unit (Achieva, Philips Healthcare Systems, Best, Netherlands) with a dedicated elbow coil was used to perform all MR imaging. Imaging was performed while the patients were in a supine position with the arm attached to the side of the body, in full extension of the elbow, and in full supination of the wrist. The MR imaging protocols were as follows: (1) axial fat-suppressed T2-weighted fast spin-echo (FSE), (2) axial T1-weighted FSE, (3) coronal T2-weighted FSE, (4) coronal fat-suppressed T2-weighted FSE, (5) coronal T1-weighted FSE, and (6) sagittal T2-weighted FSE (Table 1).

**Table 1:** Parameters of magnetic resonance imaging sequences

Plane	Sequence	TR (ms)	TE (ms)	FOV (mm)	Section thickness (mm)	Gap (mm)	Matrix
Axial	T2 fat-suppressed FSE	3026–3873	67–74	120 × 120	3	0.3	200 × 195
Axial	T1 FSE	635–702	8–12	120 × 120	3	0.3	300 × 261
Coronal	T2 FSE	3369–4020	68–82	140 × 140	3	0.3	352 × 298
Coronal	T2 fat-suppressed FSE	2418–3271	64–73	140 × 140	3	0.3	232 × 226
Coronal	T1 FSE	540–680	14–17	140 × 140	3	0.3	352 × 306
Sagittal	T2 FSE	2840–3564	87–104	142 × 142	3	0.3	358 × 283

*TR* repetition time, *TE* echo time, *FOV* field of view, *FSE* fast spin-echo, *T1* T1-weighted, *T2* T2-weighted

All MR images were reviewed by four specialists (two radiologic specialists and two orthopedic surgeons), who were blinded to the demographic and clinical information of the patients. Joint pathology (cartilage injury and joint effusion), collateral ligament injury (especially lateral ulnar collateral ligament injury), and muscle edema were assessed in MR images of 2D image data. The presence of each abnormality was examined using all MR imaging protocols. The MR images of each patient were evaluated a total of two times by four specialists. If all eight assessments matched, the results of the assessment were adopted. Otherwise, the presence of each abnormality was decided upon agreement by the four specialists. The inter-observer reliabilities of the assessments were evaluated by the intra-class correlation coefficient (ICC) using 2-way random effects and absolute agreement with the mean of multiple measurements model (ICC [2, k]), and the intra-observer reliabilities were evaluated by ICC using 2-way random effects and absolute agreement with the single measurement model (ICC [1, 2]). For intra-observer reliability, the presence of each abnormality was evaluated three times at 2 weeks interval to ensure that the assessments were independent.

### 3D reconstruction of the common extensor tendon

**Fig. 1:**
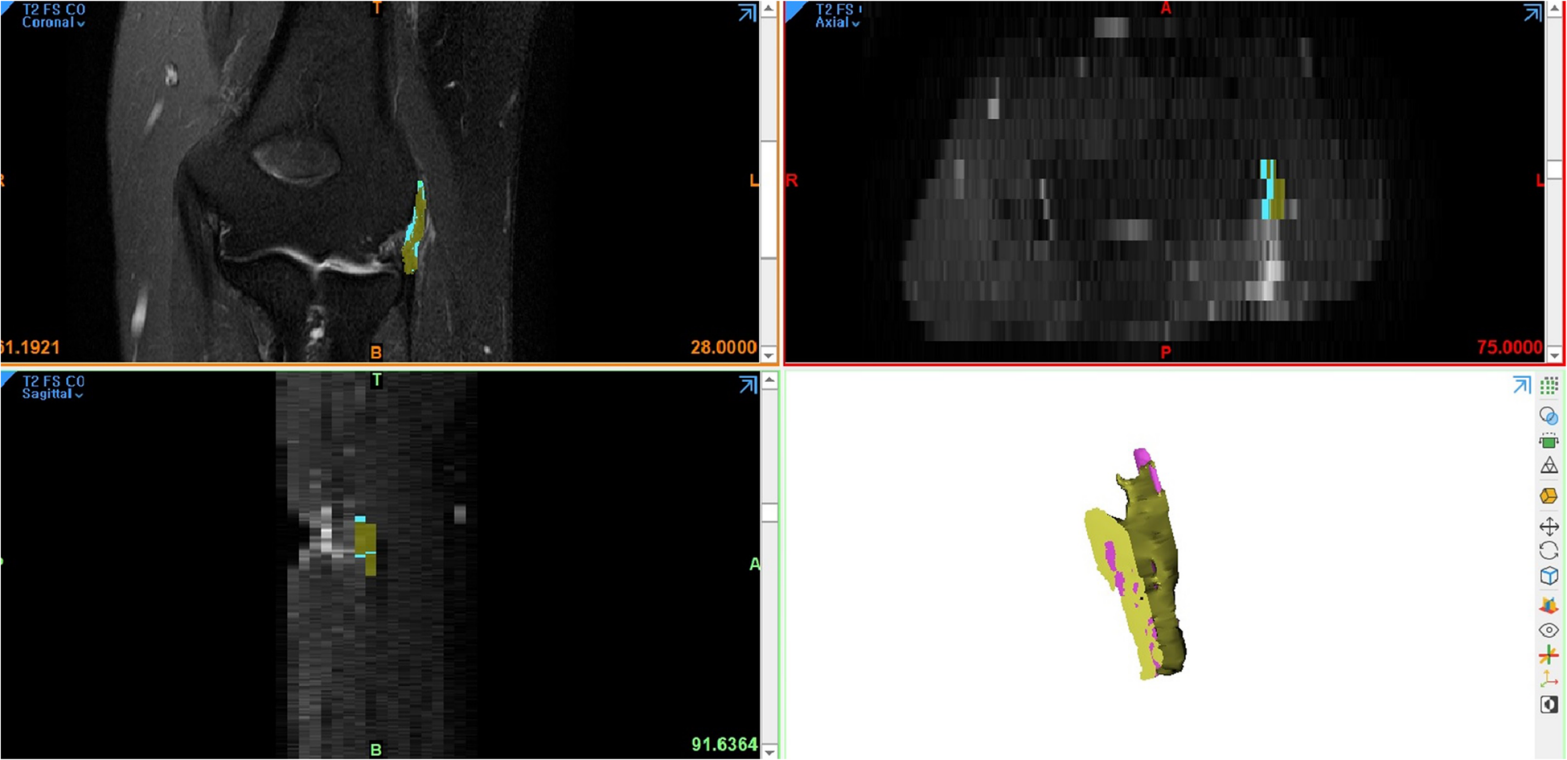
A screenshot of the three-dimensional (3D) graphic processing software. Digitized magnetic resonance imaging data in DICOM format were imported and the coronal, sagittal, and axial views of the computed tomography data were obtained in 3D graphic processing software (Mimics® 22.0, Materialise, Antwerp, Belgium)

**Fig. 2:**
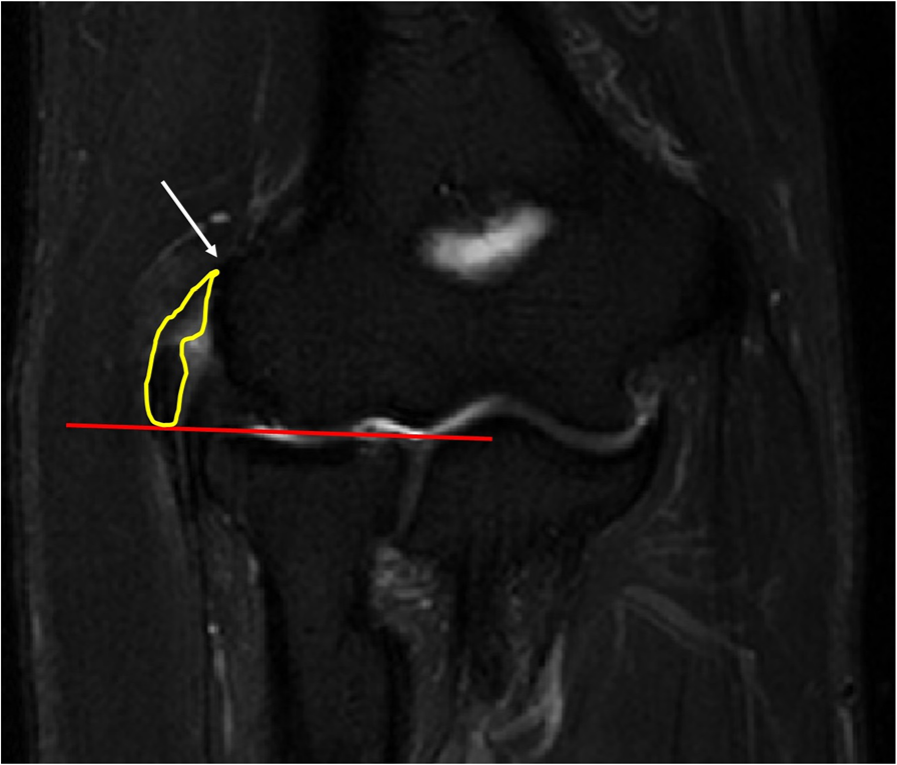
The proximal and distal boundaries of the common extensor tendon on the coronal fat-suppressed T2-weighted magnetic resonance imaging. The white arrow indicates the insertion site of the common extensor tendon located in the lateral humeral epicondyle, and the red line indicates the articular surface level of the radial head. A yellow-colored closed curve shows the margin of the region of interest of the common extensor tendon

**Fig. 3:**
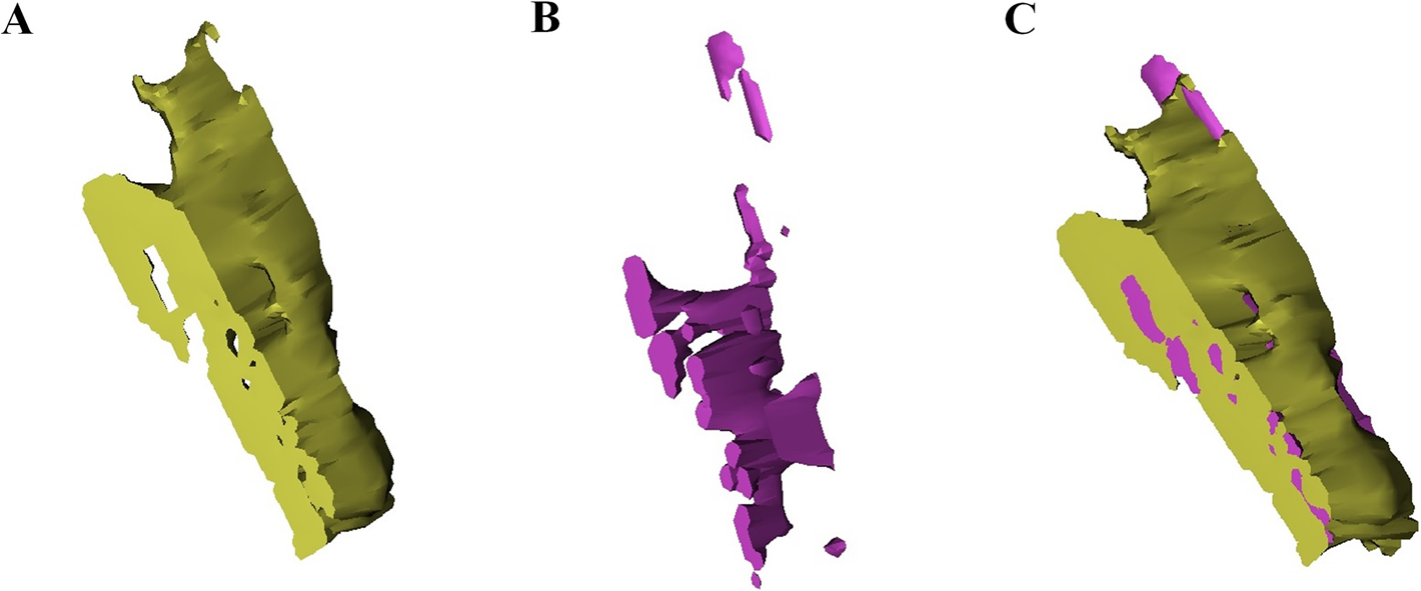
Three-dimensional (3D) reconstructed region of interests (ROIs) of the common extensor tendon and the lesion of the common extensor tendon. **a** 3D reconstructed ROI of the common extensor tendon. **b** 3D reconstructed ROI of the lesion of common extensor tendon. (C) 3D reconstructed ROIs of the common extensor tendon and the lesion of common extensor tendon

**Fig. 4:**
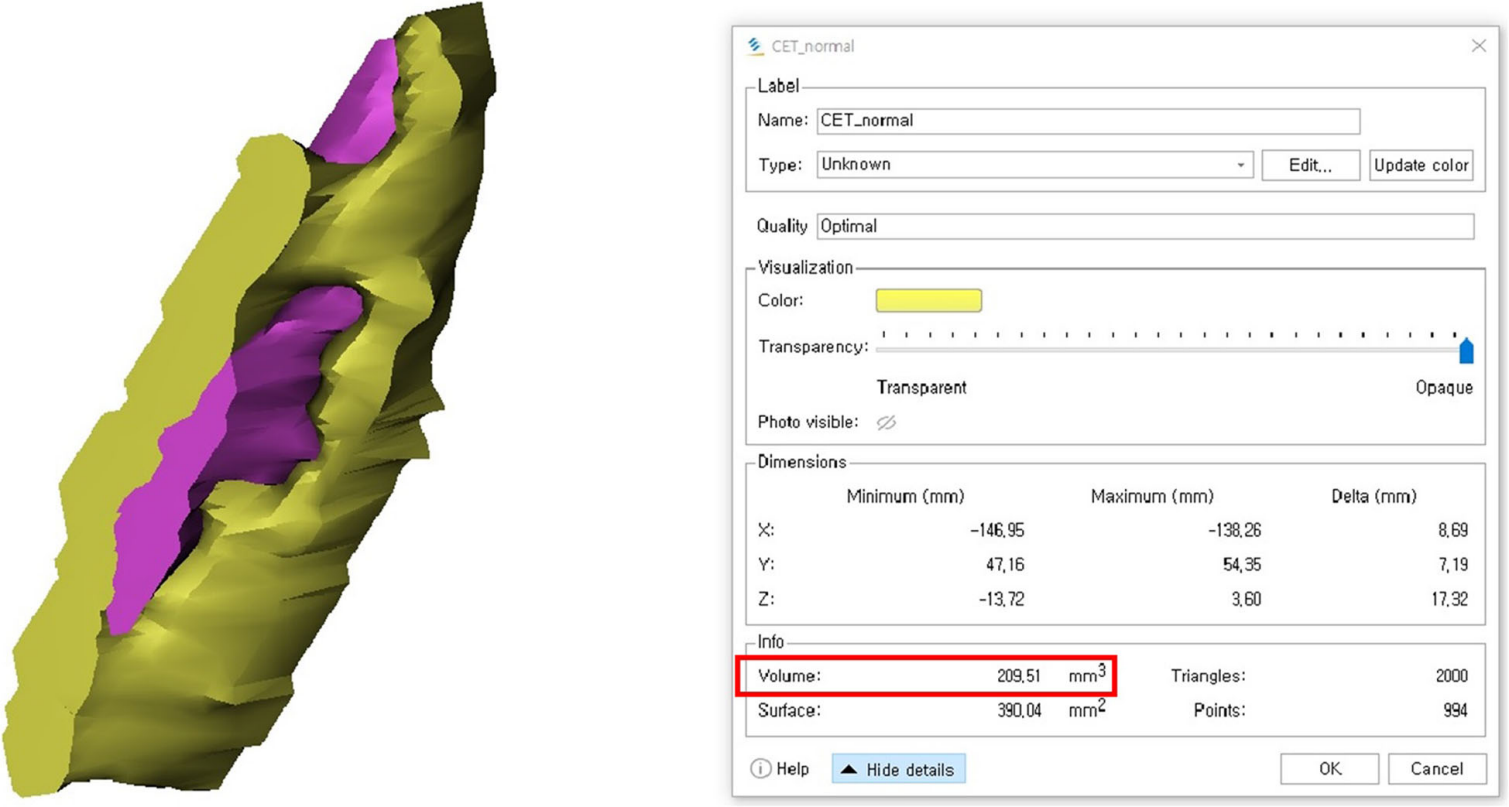
A screenshot showing the calculation process of the volume of the region of interest (ROI). The volume of ROI was calculated automatically in Mimics® software

The digitized MR imaging data in the Digital Imaging and Communications in Medicine (DICOM) format files were imported into 3D reconstruction modeling software (Mimics® 22.0, Materialise, Antwerp, Belgium) (Fig. 1). Fat-suppressed T2-weighted FSE sequence images were adopted for 3D reconstruction because those sequences are considered suitable for the evaluation of tendon pathology such as tendinosis and tendon tear [12]. The boundary of the common extensor tendon of each participant on the fat-suppressed T2-weighted FSE sequence was determined by agreement of the four specialists. The proximal boundary of the common extensor tendon was set as the insertion site of the common extensor tendon located in the lateral humeral epicondyle, and the distal end was set as the articular surface level of the radial head (Fig. 2). The common extensor tendon lesion boundary was defined as the area of high signal intensity, which showed a different signal intensity from the normal tendon within the boundary of the common extensor tendon. Mimics® software was used to convert voxels within the common extensor tendon boundary to density masks (Fig. 3). The voxels corresponding to the common extensor tendon lesions and normal common extensor tendon within the common extensor tendon boundary were separately converted to density masks. Density masks based on voxels within the common extensor tendon boundary were defined as region of interests (ROIs) of common extensor tendon, and density masks based on voxels of common extensor tendon lesions were defined as ROI of common extensor tendon lesion. Mimics® software was also used to automatically calculate the volume of ROI of common extensor tendon and the volume of ROI of common extensor tendon lesion (Fig. 4). The proportion occupied by the volume of ROI of common extensor tendon lesion in the volume of ROI of common extensor tendon was defined as the proportion of lesion volume of common extensor tendon (pLCET). pLCET values were calculated for each participant and used for analysis.

### Measurements of clinical parameters

Because pain is accounted for the majority symptom of lateral epicondylitis, the pain VAS score can be regarded as an indicator of clinical status in patients with lateral epicondylitis.^7^ QuickDASH is a representative patient-reported outcome measurement to evaluate the extent of subjective discomfort and functional performance of the upper limb [13], and ROM of joint reflect the objective function of extremities. Therefore, in this study, pain VAS score, QuickDASH score, and elbow ROM were adopted for clinical parameters.

All clinical parameters were evaluated two weeks before MR imaging was performed. The patients were asked to assess lateral epicondylitis-related pain using a VAS ranging from 0 to 10, with higher scores representing more severe pain. QuickDASH is one of the most widely used self-reporting questionnaires for upper extremity functional performance, and its validity and reliability were verified in various upper extremity diseases [13]. Eleven items of disability/symptom scale of the QuickDASH questionnaire excluding two-optional modules were used. Using a goniometer (NutriActiva, Minneapolis, Minnesota, USA), elbow ROM was measured while the patients were in the supine position. First, the flexion contracture and forward flexion of the affected elbow joint were measured. The elbow ROM was calculated as the difference between forward flexion and flexion contracture. All patients were positioned with the shoulder in neutral flexion and abduction and the forearm in full supination. The goniometer was aligned as follows: (1) the proximal arm was positioned parallel to the lateral midline of the humerus, (2) the distal arm was positioned parallel to the lateral midline of the radius, and (3) the center of the goniometer was located at the lateral epicondyle of the humerus [14]. Elbow ROM was measured by one orthopedic surgeon (EK), and the intra-observer reliability of the goniometric measurements was evaluated by measuring the elbow full forward flexion in all participants. The intra-observer reliability of the goniometric measurements at 1-month intervals was evaluated using the ICC (2, 1) to ensure that the measurements were independent.

### Statistical analysis

A Shapiro-Wilk normality test revealed that the data from the present study were normally distributed. Therefore, parametric tests were performed. A power analysis indicated that a sample of 61 participants for a multiple linear regression with three main predictors would provide 90% statistical power and 0.05 significance level with a medium to large effect size (*f*^2^ = 0.25). Univariate linear regression analyses were used to evaluate the relationship between each clinical outcome (pain VAS score, QuickDASH score, and elbow ROM) and each independent variable (demographic factors, MR comparisons, and pLCET). Each variable with a significant outcome in the univariate linear regression analysis (*P* < 0.10) was integrated into the multivariate linear regression to determine associated factors of clinical outcomes. In the multivariate linear regression analysis, forward stepwise variable selection was used, and the marginal significance levels for the entry and removal were established at 0.05 and 0.1, respectively. Statistical significance was set at *P* < 0.05. SPSS software (ver. 24.0; SPSS Inc., Chicago, IL, USA) was used to perform all statistical analyses.

## Results

### Demographic and clinical parameters and analytical values of 2D and 3D MR imaging

At the time of undergoing MR imaging, the mean age of the patients was 48.5 ± 10.3 years (range: 18–70 years), and the mean BMI was 24.6 ± 2.7 (range: 19.6–29.3) (Table 2). Eighteen patients had muscle edema and 14 patients had joint effusion. Additionally, 16 patients had collateral ligament injury, 3 patients had cartilage injury, and 6 patients had ulnar nerve problem (Table 3). The mean pLCET was 14.70 ± 9.88% (range: 0.87%– 43.63%) (Table 3). Table 4 shows the ICC (2, k) for inter-observer reliability of the abnormalities evaluated using 2D MR imaging and the ICC (2, 1) for intra-observer reliability of each specialist. The ICC (2, 1) for intra-observer reliability of the goniometric measurements was 0.96. Therefore, the inter- and intra-observer reliability of 2DMR imaging evaluations and intra-observer reliability of the goniometric measurements were considered acceptable.

**Table 2:** Demographic and clinical characteristics of the patients included in the present study

Characteristics	Number or score
Participants	61
Mean age at MR imaging taken (year)	48.51 ± 10.27 (18 – 70)
Gender (men/women)	24 (39.3%) / 37 (60.7%)
Affected sides (right/left)	37 (60.7%) / 24 (39.3%)
Body mass index (kg/m^2^)	24.64 ± 2.70 (19.59–29.3)
Pain VAS score	5.36 ± 1.76 (1–9)
QuickDASH score	44.19 ± 21.67 (4.55–86.36)
Elbow active flexion contracture (degrees)	4.34 ± 4.87 (0–20)
Elbow active forward flexion (degrees)	121.80 ± 11.03 (90–135)

Descriptive values are shown as mean ± standard deviation (range) or number of cases (proportion (%))

*MR* magnetic resonance, *VAS* wisual analog scale, *QuickDASH* Quick Disabilities Arm, Shoulder, and Hand questionnaire

**Table 3:** Two- and three-dimensional magnetic resonance image analytical values

Characteristics	Number or score
Muscle edema (yes / no)	18 (29.5%) / 43 (71.5%)
Joint effusion (yes / no)	14 (23.0%) / 47 (77.0%)
Collateral ligament injury (yes / no)	16 (26.2%) / 45 (73.8%)
Cartilage injury (yes / no)	3 (5.0%) / 58 (95.0%)
Ulnar nerve neuritis (yes / no)	6 (9.8%) / 55 (90.2%)
Volume of common extensor tendon	442.68 ± 98.07 (221.24–592.59)
Volume of common extensor tendon lesion	66.53 ± 51.33 (3.62–247.17)
pLCET (%)	14.70 ± 9.88 (0.87–43.63)

Descriptive values are shown as mean ± standard deviation (range) or number of cases (proportion (%))

*pLCET* proportion of lesion volume of common extensor tendon

**Table 4:** Inter-observer and intra-observer reliabilities of two-dimensional magnetic resonance image measurements

Items	ICC (2, 1) for Intra-observer reliability				ICC (2, k) for Inter-observer reliability
	Observer 1	Observer 2	Observer 3	Observer 4	
Muscle edema	0.943	1.000	0.884	0.943	0.788
Joint effusion	0.940	0.872	0.898	0.955	0.863
Collateral ligament injury	0.979	0.837	0.931	0.936	0.890
Cartilage injury	0.919	1.000	0.961	0.919	0.813
Ulnar nerve neuritis	1.000	0.853	0.832	0.956	0.966

ICC (2, 1), intra-class correlation coefficient (ICC) using 2-way random effects and absolute agreement with the single measurement model; ICC (2, k), ICC using 2-way random effects and absolute agreement with the mean of multiple measurements model

### Associations between clinical outcomes and the independent variables

Univariate analyses showed that sex (*P* = 0.028), muscle edema (*P* = 0.045), and pLCET (*P* = 0.092) were significantly associated with pain VAS score. However, no independent variable was associated with QuickDASH score and elbow ROM (Table 5). The three variables were included in a multivariate linear regression analysis of pain VAS score, which revealed that an increase of pain was associated with female gender (*P* = 0.025) and a higher degree of muscle edema (*P* = 0.040). However, pLCET was not associated with pain severity (Table 6).

**Table 5:** Univariate linear regression analysis of factors related to clinical parameters

Associated factors	Regression coefficient	Standard error	95% Confidence interval	*P* value
**Dependent variable: pain VAS score**				
Age	0.003	0.022	(- 0.041, 0.048)	0.883
Gender	- 1.007	0.447	(- 1.900, - 0.113)	0.028*
Affected side	0.045	0.465	(- 0.886, 0.976)	0.923
Body mass index	- 0.032	0.085	(- 0.202, 0.138)	0.708
Muscle edema	0.986	0.482	(0.022, 1.950)	0.045*
Joint effusion	- 0.005	0.541	(- 1.087, 1.077)	0.993
Collateral ligament injury	0.358	0.515	(- 0.672, 1.388)	0.489
Cartilage injury	0.322	1.051	(- 1.780, 2.424)	0.760
Ulnar nerve neuritis	0.155	0.763	(- 1.373, 1.682)	0.840
pLCET	0.039	0.023	(- 0.007, 0.084)	0.092*
**Dependent variable: QuickDASH score**				
Age	0.400	0.270	(- 0.140, 0.940)	0.143
Gender	- 3.688	5.707	(- 15.109, 7.733)	0.521
Affected side	1.033	5.726	(- 10.425, 12.491)	0.857
Body mass index	0.891	1.040	(- 1.190, 2.972)	0.395
Muscle edema	4.126	6.111	(- 8.103, 16.355)	0.502
Joint effusion	0.170	6.654	(- 13.144, 13.484)	0.980
Collateral ligament injury	- 1.747	6.357	(- 14.467, 10.973)	0.784
Cartilage injury	5.317	12.921	(- 20.531, 31.171)	0.682
Ulnar nerve neuritis	- 9.939	9.306	(- 28.560, 8.682)	0.290
pLCET	0.378	0.281	(- 0.185, 0.941)	0.184
**Dependent variable: Elbow ROM**				
Age	0.009	0.165	(- 0.320, 0.339)	0.955
Gender	4.533	3.383	(- 2.238, 11.303)	0.185
Affected side	2.680	3.417	(- 4.157, 9.517)	0.436
Body mass index	- 0.080	0.627	(- 1.336, 1.175)	0.899
Muscle edema	- 4.671	3.628	(- 11.930, 2.589)	0.203
Joint effusion	0.980	3.988	(- 6.999, 8.960)	0.807
Collateral ligament injury	2.597	3.799	(- 5.005, 10.199)	0.497
Cartilage injury	- 2.586	7.751	(- 18.097, 12.924)	0.740
Ulnar nerve neuritis	6.515	5.570	(- 4.630, 17.660)	0.247
pLCET	0.046	0.171	(- 0.296, 0.388)	0.789

^*^P < 0.1 by Univariate linear regression analysis

*VAS* visual analog scale; *pLCET* proportion of lesion volume of common extensor tendon, *ROM*, range of motion

**Table 6:** Multivariate linear regression analysis of factors related to pain visual analog scale (VAS) score

Dependent variable: Pain VAS score (*R*^2^ = 0.145, *P* value = 0.040)					
Associated factors	Unadjusted		Standardized		***P*** value
	***B***	SE	***β***	t	
Gender	- 1.001	0.434	- 0.280	- 2.306	0.025*
Muscle edema	0.979	0.465	0.256	2.106	0.040*
pLCET			0.053	0.362	0.719

^*^P < 0.05 by Multivariate linear regression analysis

*VAS* visual analog scale; *pLCET* proportion of lesion volume of common extensor tendon

## Discussion

The novel finding of the present study was lack of association between 3D reconstructed pLCET and clinical parameters. The aforementioned results showed that female gender and increased severity of muscle edema were independently associated with pain severity. However, there was no association between pLCET and pain severity. In patients with lateral epicondylitis, the clinical symptoms appeared to be affected by factors other than the volume of common extensor tendon lesion. The pain intensity can be affected by various factors, including the patient’s pain threshold and psychological traits [2]. Moreover, although the effect of gender on pain related to musculoskeletal disorders is debatable, women appear to have higher sensitive pain susceptibility then men [15, 16]. Muscle edema generally accompanies with increased local blood flow and inflammatory responses, which may lead to the activation of nociceptors in muscles [17, 18]. Therefore, despite the size of the common extensor tendon lesion being same, patients could suffer from different levels of pain severities.

The results of this study showed that the QuickDASH score and elbow ROM were not associated with demographic factors and MR comparisons. This may imply that functional status of the upper extremity might not be affected by anatomic abnormalities or demographic factors in patients with lateral epicondylitis. ROM limitation of the elbow typically occurs in the late stage of lateral epicondylitis and may not be detectable in MR imaging [2, 19]. In addition, there may be a discrepancy between the degree of common extensor tendon abnormality and the QuickDASH score because the patient’s subjective factors could affect the QuickDASH score. Therefore, care must be taken to interpret the functional status in patients with lateral epicondylitis.

Lateral epicondylitis is one of the most prevalent disease, causing lateral elbow pain, and it is difficult to control the symptoms due to the complex elbow structures and diverse pathophysiology [20]. Generally, lateral epicondylitis may arise from the functional limitation of the affected side of the upper limbs during the long treatment period, and this can ultimately lead to diminished quality of life [21]. To overcome this functional limitation and decrease the size of common extensor tendon lesions, several treatment modalities have been suggested. However, no consensus of standardized treatment protocols has been attained, and few diagnostic markers have been proposed to predict treatment outcomes [1, 6, 7]. Many previous studies focused on the association between the size of common extensor tendon lesion and clinical symptoms. However, there were some limitations in these studies; most adopted 2D imaging analysis to evaluate the size of common extensor tendon lesion.

With the recent advancement of 3D graphic processing technology, soft tissue images obtained using conventional MR imaging can be easily reconstructed to 3D models [22]. The volume of the soft tissue and the accurate location of the lesion, which is difficult to evaluate in 2D images, can be determined. In addition, owing to introduction of advanced MR imaging machine, which has an excellent spatial resolution, precise reconstruction of soft tissues becomes possible [23]. Therefore, 3D reconstruction method used in this study may improve diagnosis and management of musculoskeletal disorders including lateral epicondylitis.

This study had several limitations. First, because the mean thickness of common extensor tendon is approximately 4–5 mm [24], the common extensor tendon ROI reconstructed using the 3-mm thick section in MR imaging may not reflect the complete common extensor tendon structure. Second, although the common extensor tendon boundary was determined by the agreement of four experts, the result may differ from the actual common extensor tendon boundary. Third, the common extensor tendon lesions defined in 3D MR imaging included various tissue abnormalities that showed an altered tendon signal. Therefore, the difference in clinical manifestations according to the type of tissue abnormalities could not be considered.

## Conclusions

The aforementioned results showed that 3D reconstructed pLCET was not significantly associated with clinical parameters, and several factors might influence the degree of pain and function of the elbow. These factors, including the patient’s pain susceptibility and gender should be considered for the management of patients with lateral epicondylitis. In addition, this 3D reconstruction method might be useful for 3D MR imaging analysis.

## Data Availability

The datasets used and analyzed during the current study area available from the corresponding author on reasonable request.

## Abbreviations

MR: Magnetic resonance
ROM: Range of motion
2D: Two-dimensional
3D: Three-dimensional
CT: Computed tomography
BMI: Body mass index
VAS: Visual analog scale
QuickDASH: Quick Disabilities of Arm, Shoulder, and Hand
FSE: Fast spin-echo
ICC: Intra-class correlation coefficient
DICOM: Digital imaging and communications in medicine
ROI: Region of interest
pLCET: Proportion of lesion volume of common extensor tendon
